# Baseline low ALT activity is associated with increased long-term mortality after COPD exacerbations

**DOI:** 10.1186/s12890-020-1169-z

**Published:** 2020-05-11

**Authors:** N. Lasman, M. Shalom, N. Turpashvili, G. Goldhaber, Y. Lifshitz, E. Leibowitz, G. Berger, G. Saltzman-Shenhav, A. Brom, D. Cohen, C. Avaky, G. Segal

**Affiliations:** 1 Internal medicine T, Chaim Sheba Medical Center, Tel-Hashomer, 2nd Sheba road, Ramat Gan, Israel; 2 Sackler faculty of medicine, Tel-Aviv University, Tel-Aviv, Israel; 3 Department of Internal Medicine “A”, Yoseftal Hospital, Eilat, Israel; 4 Department of Internal Medicine “B”, Rambam Health Care Campus, Haifa, Israel; 5 Rappaport’s Faculty of Medicine, The Technion Institute, Haifa, Israel; 6 Division of Pulmonary Medicine, Rambam Health Care Campus, Haifa, Israel

**Keywords:** ALT, COPD, Exacerbation, Frailty, Sarcopenia, Survival

## Abstract

**Background:**

COPD exacerbations have negative impact on patients’ survival. Several risk factors for grave outcomes of such exacerbations have been descried. Muscle dysfunction and mass loss were shown to impact negatively on prognosis and survival. Low activity of the enzyme ALT (Alanine amino-transferase) in the blood is a known indicator for sarcopenia and frailty, however, no previous studies addressed the association of low ALT amongst patients hospitalized due to COPD exacerbation and long-term survival.

**Methods:**

This is a historic prospective cohort study of patients hospitalized due to acute COPD exacerbation.

**Results:**

Included were 232 consecutive COPD exacerbation patients. The median time of follow-up was 34.9 months (IQR 23.13–41.73 months). During this period 104 (44.8%) patients died. All patients were grouped to quartiles according to blood ALT levels (after exclusion of cases considered to have hepatic tissue damage (ALT > 40 IU)). The risk of long-term mortality increased, in a statistically significant manner, amongst patients with low ALT values: the median survival of patients with ALT < 11 IU was 18.5 months only while the median survival for the rest of the study group was not reached. For ALT < 11 IU; 12-16 IU; 17-20 IU and > 21 IU the mortality rates were 69%; 40.9%; 36.3 and 25% respectively (*p* <  0.001 for comparison of lower quartile with upper three quartiles). The crude hazard ratio for mortality amongst patients with ALT levels lower than 11 IU was 2.37 (95% CI; 1.6–3.5). This increased risk of mortality remained significant after adjustment for age, weight, creatinine, albumin concentration and cardiovascular diseases (HR = 1.83; 95% CI 1.08–3.1, *p* <  0.05).

**Conclusions:**

Low ALT values, a biomarker of sarcopenia and frailty, are associated with poor long-term survival amongst patients hospitalized due to COPD exacerbation.

## Background

Chronic obstructive pulmonary disease (COPD) is a highly prevalent disease worldwide. Acute COPD exacerbations have dismal results, both in high patients’ mortality (ranging in different publications from over 5% to over 18% in-hospital mortality during exacerbation [1, 2]) and in extremely large health expenses [1–6]. Several risk factors for grave outcomes of COPD exacerbations have been described: Sakamoto et al. [6] performed multivariable logistic regression analysis of over 3000 COPD patients and found that older age and lower body mass index, amongst other patients’ characteristics, are significantly associated with increased risk of in-hospital mortality. Serra-Picamal et al., in a big-data analysis of 17,555 COPD patients, identified early re-hospitalizations as another risk factor for mortality after hospitalization due to COPD exacerbation [7].

Survival and COPD mortality are influenced by both dysfunction and impaired muscle mass (sarcopenia) [8, 9]. Additionally, COPD exacerbations also rapidly induce loss of muscle mass and function through the activation of several biological pathways and systems. Sarcopenia (decreased muscle mass associated with increased risk of hospitalizations, falls and mortality) and frailty (increased tendency to succumb to morbidities, often associated with sarcopenia), two overlapping syndromes, defined as causing increased mortality and frequent hospital admissions, are prime candidates for having a negative impact on COPD exacerbations’ outcomes. In a review published back in 2005, Morley et al. [10] stated that COPD is one of the chronic diseases particularly associated with frailty. Vellas et al. [11] relate to COPD as a causative agent for decreased muscle mass, in turn becoming a risk factor for COPD patients’ mortality. In their review regarding “Sarcopenia and frailty in chronic respiratory disease”, Bone et al. [12] declare the association between frailty and poor outcomes amongst COPD patients. Maddocks et al. [13] found that frailty affect 25% of all COPD patients and that it is a significant factor for pulmonary rehabilitation failure. Nevertheless, they state that pulmonary rehabilitation programs are beneficial for frail, COPD patients, potentially reversing frailty characteristics even with a short-term rehabilitation program.

Low ALT activity in the peripheral blood is associated with low, total-body, muscle mass. This is specifically accurate when there is no spillage of ALT from hepatocytes to the peripheral blood, such as in cases of hepatitis. Therefore, in the current study, as done in previous studies, patients with higher than normal ALT values were excluded from analysis. Previous studies have demonstrated a significant correlation between ALT values and muscle mass, as demonstrated by computed tomography measurements [14] and between low ALT and frailty, as appreciated by the FRAIL questionnaire [15]. Other studies have showed that low ALT levels are associated with long term all-cause mortality in the elderly population [16–19]. Sarcopenia and the resultant frailty, represented by low activity of the enzyme ALT (SGPT) in the peripheral blood, were shown to be associated with shortened survival and other poor clinical outcomes, in various patients’ populations and clinical scenarios. It is postulated that this association rise from the fact that low ALT levels are the result of sarcopenia and the resultant frailty is the cause for shortening of life span in low-ALT populations [20–23].

However, there are no previous studies that examined the relationship between low ALT values and mortality amongst hospitalized patients with COPD exacerbation. The aim of the current study was to assess the association between ALT and clinical outcomes.

## Patients and methods

### Patient selection

We executed a historic, prospective cohort study of COPD patients hospitalized to a single department of internal medicine, due to COPD exacerbation as their main diagnosis. The inclusion criteria were: a. age over 18 years, b. diagnosis of COPD exacerbation (ICD code 496) on discharge, c. available ALT blood levels, within the normal limits (lower than 40 IU/L) during the 3 months prior to admission (in-hospital ALT levels, normally ranging from 7 to 45 IU/L, were tested using a standard Beckman Coulter®. Quantitative determination of ALT levels was used by applying kinetic UV tests. In order to assure maximal catalytic activity of the ALT from the blood samples, test tubes were supplemented with activated pyridoxal phosphate (P-5-P), serving as an essential co-factor for ALT catalytic activity. There are no significant normal-range differences between in- and out-of-hospital ALT measurement methods). Over 95% of ALT baseline values were available from in-hospital measurements while a small minority of ALT values were extracted from pre-hospitalization records. After study approval by an institutional ethics review board, we excluded patients with ALT levels greater than 40 IU/L, supposed to have hepatic tissue damage of any cause. We extracted patients’ details from the computerized records, including epidemiologic characteristics, background diagnoses, long-term medications, laboratory parameters, short- and long-term clinical outcomes. Data extraction from patients’ computerized records was made by two researchers who went through all “free text” contents of these records. Mortality data was extracted from the Israeli national death certificates registry. The Norton scale, a score that is used by nurses around the world since the early 1960s to assess the risk of pressure ulcers upon admission and during hospitalization, was extracted too from the medical record. It includes five domains, each is a fundamental aspect of health in the elderly: physical condition, mental condition, activity, mobility, and incontinence [24].

### Statistical methods

In order to assess the association between ALT levels and dichotomous variables we calculated the sample size, as appropriate for medium strength associations. In order to determine such association, we needed a sample size greater than 192 patients (for 1% significance and 80% power). The cohort size was determined using G Power software [25].

We assessed the distribution of continuous variables using histograms and Q-Q plots. We described continuous variables with normal distribution by means and standard deviations. Continuous variables that did not have normal distribution were described using median and IQR (Inter-quartile range). We described categorical variables using frequency and percentages. ALT level was divided into two categories using the lower quartile as a cut off value for categorization. We assessed the association between ALT category and continuous variables using t-tests or Mann-Whitney tests. We assessed the association with categorical variables using the chi-square tests or the exact fisher’s test. Kaplan-Meier curve was used to describe survival during the follow up period and log-rank test was used to compare between ALT categories. Univariate Cox regression was used to study the association between the various parameters and mortality. We used Cox regression to assess the association between ALT category while controlling for possible confounders. Statistical analysis was done using the SPSS software (IBM Corp., NY USA). All statistical tests were two-sided. We considered as significant only *p* values lower than 0.05.

## Results

**Fig. 1:**
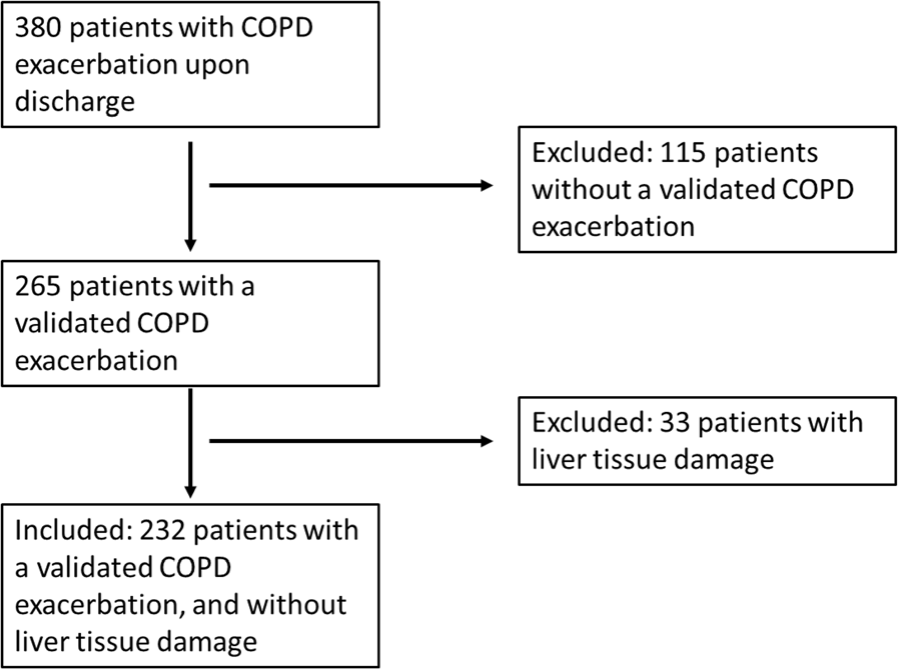
Consort diagram of patients’ flow in the current study

We included in this study 232 consecutive COPD patients, hospitalized due to COPD exacerbation in one internal-medicine department in a large, tertiary hospital, over a period of 38 months – from January 2015 to February 2018. After approval by an IRB, we extracted patients’ data from their computerized medical records. The mean age was 76.06 ± 10.7 years, median Norton score was 15.7 (IQR 13–19), median ALT level was 16.5 IU/L (IQR 11–20), median creatinine concentration was 1.15 mg/dL (IQR 0.74–1.35), and the mean albumin concentration was 3.6 ± 0.47 g/dL. Within our cohort, 74.6% had cardio-vascular diseases, 59.9% had metabolic diseases, 35.3% had diabetes, 25.4% had neurological diseases, and 24.1% suffered from hematological or hemato-oncological diseases. Regarding background medical treatments, 19.4% were treated by systemic steroids, 32.8% took anti-diabetic medications, and 84.1% had cardiovascular medications, 26.3% treated by anticoagulants and 67.7% were using inhalers regularly. Patients’ characteristics, according to their baseline ALT values (lower quartile (< 11 IU/L) vs. upper three quartiles (11 IU/L < ALT < 40 IU/L)), are described in Table 1. The median time of follow-up was 34.9 months (IQR 23.13–41.73). During this period 104 (44.8%) patients died. Figure 1 describe the flow of patients from recruitment to analysis and Table 2 describe patient characteristics found to be associated with increased risk for mortality amongst our study cohort.

**Table 1:** Patients’ characteristics according to baseline ALT levels

	ALT < 11 IU/L	11 IU/L ≤ ALT ≤ 40 IU/L	***P*** value
**Patients’ demographics**			
Age (years ± SD)	78.8 ± 9	74.8 ± 11.2	**0.008**
Gender – males (n (%))	39 (54.9)	77 (47.8)	0.32
Norton score (number, IQR)	14.93 (13–17)	16.06 (13.5–19)	**0.002**
**Background diagnoses (groups)**			
Cancer (n (%))	20 (28.2)	36 (22.4)	0.34
Dementia (n (%))	3 (4.2)	7 (4.3)	0.96
Diabetes (n (%))	33 (46.5)	49 (30)	**0.018**
Cardiovascular (n (%))	54 (76.1)	119 (73.9)	0.73
Metabolic (n (%))	47 (66.2)	92 (57.1)	0.19
Neurologic (n (%))	17 (23.9)	42 (26.1)	0.73
Endocrine (n (%))	12 (16.9)	21 (13.0)	0.49
**Medications (groups)**			
Steroids (n (%))	17 (23.9)	28 (17.4)	0.24
Anticoagulation (n (%))	27 (38)	34 (21.1)	**0.007**
Inhalers/Inhalation (n (%))	46 (64.8)	111 (68.9)	0.53
Anti-diabetic (n (%))	28 (39.4)	48 (29.8)	0.15
Cardiovascular (n (%))	62 (87.3)	133 (82.6)	0.37
Metabolic (n (%))	17 (23.9)	33 (20.5)	0.56
Neurologic (n (%))	33 (46.5)	62 (36.5)	0.25
**Laboratory parameters**			
Albumin (g/dL ± SD)	3.4 ± 0.45	3.7 ± 0.45	**< 0.001**
Creatinine (mg/dL, (IQR))	1.32 (0.93–1.53)	1.07 (0.68–1.29)	**< 0.001**
**Clinical outcomes**			
Mortality (n (%))	49 (69.0)	55 (34.2)	**< 0.001**
Survival time – median (months)	18.5	Median not reached

**Table 2:** Patients’ characteristics according to survival – univariate analysis

Patients’ characteristics	Alive	Dead	Hazard ratio (95% CI)	***P*** Value
Age (years ± SD)	72.89 ± 10.8	79.96 ± 9.2	1.06 (1.04–1.08)	**< 0.001**
Norton score (number, (IQR))	16.84 (15–19)	14.33 (11.25–17)	0.88 (0.84–0.92)	**< 0.001**
Albumin (mg/dL ± SD)	3.69 ± 0.47	3.48 ± 0.45	0.48 (0.34–0.7)	**< 0.001**
Creatinine (mg/dL, (IQR))	1.03 (0.69–1.21)	1.29 (0.84–1.62)	1.57 (1.23–2)	**< 0.001**
ALT (IU/L, IQR)	18.71 (13–23.75)	13.77 (9–17)	0.93 (0.9–0.96)	**< 0.001**
ALT < 11 IU/L (n (%))	22 (17.2%)	49 (47.1%)	2.48 (1.67–3.66)	**< 0.001**
Cardiovascular disease (n (%))	89 (69.5)	84 (80.8)	1.8 (1.1–2.93)	**0.019**
Cardiovascular medications (n (%))	101 (78.9)	94 (90.4)	2.09 (1.09–4.0)	**0.027**
Neurologic medications (n (%))	41 (32.0)	54 (51.9)	1.94 (1.31–2.86)	**0.001**
Anticoagulation medications (n (%))	29 (18.8)	37 (35.6)	1.73 (1.15–2.57)	**0.008**

**Fig. 2:**
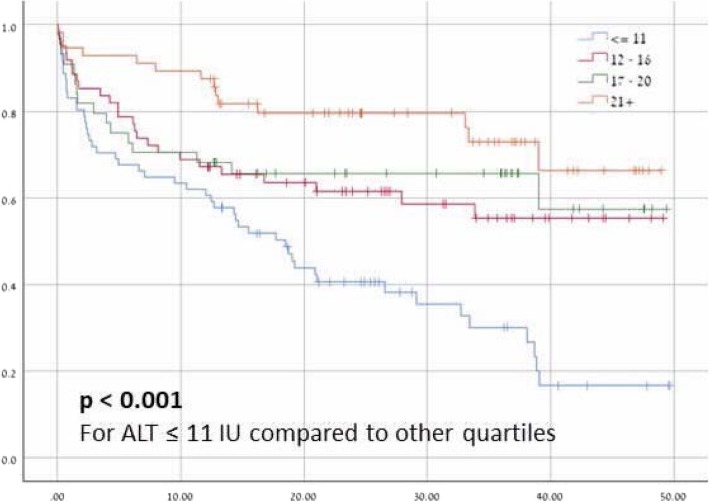
Survival (months) of COPD patients according to baseline ALT values

We divided all patients to quartiles according to their blood ALT levels (after exclusion of cases considered to have hepatic tissue damage (ALT > 40 IU)). The risk of long-term mortality increased, in a statistically significant manner, amongst patients with low ALT values: the median survival for patients with ALT < 11 IU was 18.5 months only while the median survival for the rest of the study group was not reached. For ALT < 11 IU; 12-16 IU; 17-20 IU and > 21 IU the mortality rates were 69%; 40.9%; 36.3 and 25% respectively (*p* <  0.001 for comparison of lower quartile with upper three quartiles; Fig. 2).

The crude hazard ratio for mortality amongst patients with ALT levels lower than 11 IU was 2.37 (95% CI; 1.6–3.5). This increased risk of mortality remained significant after controlling for age, weight, Norton score, creatinine, albumin concentration and cardiovascular diseases (HR = 1.83; 95% CI 1.08–3.1, *p* = 0.025). Table 3 describe the multivariate Cox regression for mortality in our study cohort.

**Table 3:** Multivariate Cox regression for mortality in our study cohort

Patients’ characteristics	Hazard ratio (95% CI)	***P*** value
Lower quartile of ALT (< 11 IU/L)	1.83 (1.08–3.1)	**0.025**
Age (years)	1.02 (0.99–1.05)	0.110
Weight (kilograms)	0.98 (0.97–0.99)	**0.02**
Norton score (number)	0.92 (0.86–0.98)	**0.016**
Creatinine value (mg/dl)	1.79 (1.14–2.81)	**0.011**
Albumin concentration (mg/dl)	0.67 (0.41–1.18)	0.178
Cardiovascular disease	1.56 (0.83–2.94)	0.167

## Discussion

As stated earlier, ALT is a biomarker for sarcopenia and frailty [14, 15, 23]. In our study, we have showed that low ALT values, are associated with poor long-term survival amongst patients hospitalized due to COPD exacerbation. Of note, our results remained significant after controlling for parameters associated with frailty, i.e. Norton scale, albumin, etc.

Appreciation of sarcopenia and frailty enables a summation of patients’ characteristics which are associated with his or her risk for subsequent morbidity and mortality. Addressing sarcopenia and frailty in quantitative measures should, therefore, better the prognostication process for many patients’ populations. COPD patients should not be an outlier for the aforementioned declaration: these patients should be routinely evaluated for sarcopenia and frailty, and treatment strategies must take into account these patient’s characteristics. Routine assessment for sarcopenia and frailty can be done by simple ALT measurements or by phenotypic tests such as hand grip strength test of the “up and go” test.

Current literature promotes personalized medicine. However, most cases of personalized medicine are actually dealing with precise medicine: diagnostic approaches become more focused on disease and pathology rather than on the person / patient as a whole. We believe that thorough evaluation of sarcopenia and frailty should be considered as true personalized medicine.

In the current study we show that similarly to other patients’ groups, COPD patients can also be classified as sarcopenic and frail, according to their baseline ALT values. ALT measurements are a part of routine blood tests and are available worldwide for any hospitalized patient. Acknowledgment of low ALT values during COPD exacerbation could aid the attending physician in taking better clinical / therapeutic decisions (duration and route of administration of steroids, duration and type of antibiotics, promoting physiotherapy sessions and rehabilitation programs prior to patient’s discharge to the community etc.).

ALT measurement should not replace other, well established methods for sarcopenia and frailty appreciation. Nevertheless, most such evaluation tools were developed and are being used in the realm of geriatric medicine. The results of this study are applicable to the general population of COPD patients admitted to internal-medicine departments. For these reasons, we suggest that ALT values would be flagged, and diagnoses of sarcopenia and frailty should be highlighted in the discharge documents of COPD patients.

### Limitations

In the study we performed a retrospective analysis and therefore, not all confounding parameters could be addressed (such as BMI and FEV_1_ values). Moreover, while demonstrating significant statistical associations between ALT and mortality, causality could not be inferred. We performed the study on patients admitted to a single internal medicine department that might not correlate perfectly to all COPD patients that are admitted to hospitals nationally.

## Conclusion

Low ALT values are an independent marker for shortened survival of COPD patients after being hospitalized due to COPD exacerbation. Being a marker for sarcopenia and frailty, low ALT values in these patients should prompt more aggressive therapeutic efforts on behalf of the attending physician. Future, prospective, interventional studies should aim at evaluation of different therapies and rehabilitation programs, concentrated at the sarcopenic and frail COPD patients.

## Data Availability

All data is available with the authors for further study questions.

## Abbreviations

ALT: Alanine amino-transferase
SGPT: Serum glutamic pyruvic transaminase
COPD: Chronic, obstructive pulmonary disease
CI: Confidence interval
IU: International units
IQR: Inter-quartile range
Q-Q Plot: Quantile-quantile plot
